# Continuous Positive Airway Pressure Versus Mandibular Advancement Devices Impact on Cardio‐Metabolic Outcomes in Patients With Obstructive Sleep Apnea: A Systematic Review and Meta‐Analysis of Randomised Controlled Trials

**DOI:** 10.1111/jsr.70351

**Published:** 2026-06-24

**Authors:** AlMothana Manasrah, Mohammad Tanashat, Ramy Ghaly, Mohamed Abouzid, Shrouq Ramadan, Abdo‐rhman Khassawneh, Abubakar Nazir, Zaid Bataineh, Mohammed Ayyad, Mohamed Abuelazm, Basel Abdelazeem, Muhammad Imran Ali

**Affiliations:** ^1^ Department of Pulmonology and Critical Care United Health Services—Wilson Medical Center Johnson City New York USA; ^2^ Faculty of Medicine Yarmouk University Irbid Jordan; ^3^ Department of Internal Medicine University of Missouri—Kansas City Kansas City Missouri USA; ^4^ Department of Physical Pharmacy and Pharmacokinetics Poznan University of Medical Sciences Poznan Poland; ^5^ Internal Medicine Department Ain Shams University Cairo Egypt; ^6^ Internal Medicine Department Henry Ford Macomb Hospital Clinton Twp Michigan USA; ^7^ Internal Medicine Department King Edward Medical University Lahore Pakistan; ^8^ Faculty of Medicine Jordan University of Science and Technology Irbid Jordan; ^9^ Department of Medicine Rutgers New Jersey Medical School Newark New Jersey USA; ^10^ Faculty of Medicine Tanta University Tanta Egypt; ^11^ Cardiology Department West Virginia University Morgantown West Virginia USA

**Keywords:** cardiology, hypertension, sleep apnea, sleep medicine

## Abstract

Obstructive sleep apnea contributes to cardiovascular morbidity, and its treatment may mitigate this risk. Continuous positive airway pressure and mandibular advancement devices are established therapies, but their comparative cardiometabolic effects remain uncertain. We conducted a systematic review and meta‐analysis of randomised controlled trials from multiple major databases through May 2024, pooling dichotomous outcomes as risk ratios and continuous outcomes as mean differences with 95% confidence intervals using StataMP version 17. Across 14 randomised trials including 1241 patients, CPAP showed clear advantages over MAD. CPAP significantly reduced low‐density lipoprotein (MD −15.20 mg/dL; 95% CI −28.86 to −1.53), total cholesterol (MD −17.10 mg/dL; 95% CI −30.15 to −4.05) and dipping diastolic blood pressure (MD −3.12 mmHg; 95% CI −5.62 to −0.62). No meaningful differences emerged between the two therapies for serum glucose, HDL, triglycerides, 24‐h mean blood pressure, systolic or diastolic pressures during sleep or wakefulness, or heart rate metrics. Overall, CPAP demonstrated superior lipid and diastolic blood pressure improvement compared with MAD, pointing towards a more favourable cardiometabolic profile. Both interventions remain effective treatments for OSA, yet CPAP may provide added benefit in reducing cardiovascular risk factors and appears to be the preferred modality in patients with elevated cardiometabolic risk.

## 1 Introduction

Obstructive sleep apnea (OSA) is a prevalent sleep disorder marked by repeated upper airway obstruction during sleep, leading to intermittent hypoxemia and sleep fragmentation (Patil et al. 2019). Affecting nearly one billion people globally, about 425 million adults aged 30–69 experience moderate to severe OSA, with 15 or more events per hour (Pataka et al. 2023). In the United States, 25%–30% of men and 9%–17% of women meet the diagnostic criteria, with higher prevalence in Hispanic, Black and Asian populations. Its incidence rises with age and is strongly linked to obesity, which ranges from 14% to 55% (Francis and Quinnell 2021; Dissanayake et al. 2021). Genetic factors also contribute. OSA significantly increases the risk of hypertension, arrhythmias, coronary artery disease, heart failure, stroke and sudden cardiac death (Dissanayake et al. 2021). A study found nearly 50% of men and 23% of women have moderate OSA (Page et al. 2021). Additionally, up to 70% of OSA patients meet metabolic syndrome criteria, exacerbating cardiovascular risks (Pataka et al. 2023; Dissanayake et al. 2021).

The standard treatment for moderate to severe OSA is continuous positive airway pressure (CPAP), which maintains airway patency by delivering pressurised air through a mask during sleep. CPAP therapy has been shown to significantly reduce blood pressure (BP) by 2–4 mmHg, lower the risk of cardiovascular events by 25% and improve endothelial function, systemic inflammation and arterial stiffness (Patil et al. 2019). However, adherence to CPAP is a significant challenge; up to 50% of patients discontinue use within the first year due to discomfort, inconvenience, or intolerance, limiting its clinical effectiveness (Pataka et al. 2023).

Mandibular advancement devices (MADs) offer a practical alternative for these patients. MADs reposition the lower jaw to prevent airway collapse, and studies have shown that they can improve OSA symptoms, reduce systolic and diastolic BP by 2–3 mmHg and enhance quality of life (QoL) with greater acceptance and adherence rates compared to CPAP (Francis and Quinnell 2021).

While CPAP is traditionally considered more effective, recent studies suggest that the cardiovascular and metabolic outcomes of MAD are comparable to those of CPAP, particularly regarding BP reduction. However, the majority of studies comparing MAD and CPAP have been limited by small sample sizes, short follow‐up periods, exclusion of patients with severe OSA and a lack of rigorous assessment of cardiovascular outcomes such as morbidity and mortality (Dissanayake et al. 2021).

Furthermore, only a few studies have employed gold‐standard polysomnography or evaluated newer, optimally titrated MAD devices. Given the high prevalence of OSA and its substantial impact on cardiovascular and metabolic health, there is an urgent need for robust evidence comparing CPAP and MAD therapies in terms of clinically relevant outcomes, especially in patients with moderate to severe OSA who may benefit from expanded therapeutic options. This meta‐analysis aims to comprehensively evaluate the cardiovascular and metabolic effects of MAD versus CPAP, providing critical insights into their relative efficacy and informing future treatment guidelines.

## 2 Methodology

### 2.1 Protocol Registration

This systematic review and meta‐analysis adhered to the PRISMA guidelines for reporting systematic reviews (Page et al. 2021) and the *Cochrane Handbook for Systematic Reviews of Interventions* (Higgins et al. 2019). The review protocol was registered with PROSPERO under ID: CRD42024550736.

### 2.2 Data Sources and Search Strategy

A comprehensive literature search was conducted across PubMed (MEDLINE), Scopus, Web of Science (WoS), EMBASE and the Cochrane Central Register of Controlled Trials (CENTRAL) up to 14 May 2024. Search terms and strategies were adapted for each database, with detailed results outlined in Table 1.

**TABLE 1:** Summary characteristics of the included RCTs.

Study	Design	Country	Number of centres	Blinding status	Total participants	Duration of treatment	Main inclusion criteria	Primary outcome	Follow‐up duration
Barnes et al. (2004)	RCT‐crossover	Australia	2	—	104	3‐month treatment periods with a 2‐week washout between periods	Sleep clinic patients with mild to moderate OSA (AHI, 5–30/h) and healthy, adequate dentition for MAS use.	ESS	—
Dal‐Fabbro et al. (2014)	RCT‐crossover	Brazil	—	Single‐blinding	29	1 months treatment periods with a 1‐ week washout between periods	Moderate‐to‐severe OSA (AHI ≥ 20) Either gender BMI under 35 kg/m^2^ Age 25–65 years Dentition in good condition Minimum mandibular protrusion of 7 mm	Effects on: BP Oxidative stress HR variability	—
De Vries et al. (2019)	RCT	the Netherlands	3	Open‐label	85	12 months	Patients aged 18 years or older with AHI of 15 to 30/h based on PSG.	ICER/ICUR in terms of AHI reduction and QALYs after 12 months	12 months
Dissanayake et al. (2021)	RCT	Australia	1	—	92	4 weeks	Age 20 years, new diagnosis of OSA and at least two symptoms of OSA	24‐h mean arterial pressure	4 weeks
Glos et al. (2016)	RCT	Germany	1	—	84	12 weeks each	AHI of ≥ 5/h age of ≥ 18 years Severe OSA (AHI > 30/h) requiring treatment were included only if they did not demonstrate clear indication for CPAP such as a severe cardiovascular risk, e.g., myocardial infarction, stroke, atrial fibrillation, resistant hypertension, or heart failure. An essential element for inclusion of any patient was a clinical symptom complex, as well as suffering owing to lack of refreshing sleep.	Improving parameters of daytime cardiac autonomic modulation, especially BP	24 weeks
Guimarães et al. (2021)	RCT	Brazil	1	—	79	12 months	Mild OSA, any sex, age between 18 and 65 years and a BMI of ≤ 35 kg/m^2^	24‐h ambulatory BP monitoring and PAT	12 months
Lam et al. (2007)	RCT	Hong Kong	1	—	101	10 weeks	AHI > 5–40 and ESS 19 0.9 for those with AHI 5–20	—	10 weeks
Ou et al. (2024)	RCT	Singapore	3	Open label	220	—	Adults of Chinese ethnicity aged 40 years with known hypertension and at least 1 other factor for high cardiovascular risk for screening PSG.	Difference between the 24‐h mean arterial BP at baseline and 6 months.	6 months
Phillips et al. (2013)	RCT‐crossover	Australia	3	—	122	2 months	Patients with newly diagnosed OSA (AHI 10/h) Age 20 years or older Greater than or equal to two symptoms of OSA Willingness to use both treatments	24‐h mean arterial pressure	—
Schütz et al. (2013)	RCT	Brazil	1	—	45	2 months	Male patients with moderate to severe OSA and BMI less than 30 kg/m^2^	Subjective and objective sleep parameters, QoL and mood in OSA	—
E. Silva et al. (2021)	RCT	Brazil	1	Open label	79	1 year	Patients with mild OSA (an AHI score of ≥ 5 and < 15/h), age at least 18 years to maximum 65 years and BMI ≤ 35 kg/m^2^	Metabolic profile	12 months
Uniken Venema et al. (2020)	RCT	the Netherlands	1	—	31	—	—	—	10 years
Uniken Venema et al. (2022)	RCT	the Netherlands	1	—	85	12 months	Adult participants, who had been diagnosed with moderate OSA (AHI 15–30/h), based on a single‐night PSG	AHI reduction	12 months
Yamamoto et al. (2019)	RCT‐crossover	Japan	1	—	85	16 weeks total (8 for each treatment arm)	Over 20 years of age Diagnosed with OSA with an AHI of 20–40/h and supine dependency (defned as the AHI in the supine position being more than twice that in other positions, according to the definition of Joosten et al.) based on overnight PSG	Improvement in the endothelial function, indexed by the fow‐mediated dilatation	—

Abbreviations: AHI, apnea‐hypopnea index; BMI, body‐mass index; BP, blood pressure; ESS, Epworth Sleepiness Scale; HR, heart rate; ICER, incremental cost‐effectiveness ratio; ICUR, incremental cost‐utility ratios; OSA, obstructive sleep apnea; PAT, peripheral arterial tonometry; PSG, polysomnography; QALY, quality‐adjusted life‐years; QoL, quality of life; RCT, randomised control trial.

### 2.3 Eligibility Criteria and Study Selection

The eligibility criteria for this meta‐analysis were established using the PICOS framework. Studies were included if they involved adult participants (≥ 18 years) diagnosed with OSA as the population and evaluated the use of MAD as the intervention, with CPAP as the comparator. The primary outcomes assessed were 24‐h BP (mean BP, systolic BP and diastolic BP), as well as BP measurements during wakefulness and sleep (mean BP, systolic BP and diastolic BP), along with dipping patterns in systolic and diastolic BP. The mean 24‐h heart rate (HR) and HR during wakefulness and sleep were also considered. Secondary outcomes included metabolic parameters (body mass index [BMI], waist and neck circumference), biochemical markers (low‐density lipoprotein [LDL], total cholesterol, glucose, high‐density lipoprotein [HDL] and triglycerides [TGs]) and QoL measures (Epworth Sleepiness Scale [ESS] and Functional Outcomes of Sleep Questionnaire [FOSQ]). Only randomised controlled trials (RCTs) were eligible for inclusion. Studies were excluded based on the following criteria: non‐human or in vitro studies, book chapters, reviews, commentaries, letters, editorials, guidelines, studies with overlapping or duplicate datasets and publications in languages other than English.

### 2.4 Study Selection

The study selection process was conducted using the Covidence platform. After duplicate removal, five reviewers (A.K., S.R., Z.B., A.N. M.Ay. and M.A.) independently screened the records. Full texts were reviewed for eligibility criteria, and disagreements were resolved through consensus and consultation with a senior reviewer.

### 2.5 Data Extraction

A structured data extraction template was developed using a pilot‐tested Excel spreadsheet (Microsoft, USA). The spreadsheet was divided into three sections. The first section, *Study Characteristics*, captured essential details such as the study ID, year of publication, design, country of origin, number of centres, blinding status, total number of participants, intervention and control groups, treatment duration, primary inclusion criteria, primary outcomes and follow‐up duration. The second section, *Baseline Participant Data*, included demographic and clinical characteristics such as age, male gender, BMI, waist and neck circumference, comorbidities (hypertension, dyslipidemia, smoking and diabetes), QoL metrics (ESS and FOSQ), BP measurements (24‐h mean BP, systolic BP and diastolic BP; BP during wakefulness and sleep, including systolic and diastolic BP dipping patterns), HR (24‐h, during sleep and wakefulness) and laboratory results (glucose, HDL, TG, total cholesterol and LDL). The third section, *Outcome Data*, focused on post‐treatment measurements, including BMI, waist and neck circumference, QoL scores (ESS and FOSQ), BP (24‐h mean BP, systolic BP and diastolic BP; BP during wakefulness and sleep with systolic and diastolic BP dipping patterns), HR (24‐h, during sleep and wakefulness) and laboratory results (glucose, HDL, TG, total cholesterol and LDL). Data extraction was performed by five authors (A.K., S.R., Z.B., A.N. and M.A.), with any discrepancies resolved through consensus discussions involving a senior reviewer.

### 2.6 Risk of Bias Assessment

The Cochrane Risk of Bias 2 (RoB2) tool assessed study quality across five domains: outcome measurement, selective reporting, intervention deviations, missing outcome data and randomisation‐related bias. Risk of bias was conducted by (A.K., S.R., Z.B., A.N. and M.A.). Discrepancies were resolved through discussion with a senior author.

### 2.7 Statistical Analysis

Statistical modelling and visualisation were performed using R (version 2021.09), MetaInsight and Meta‐Mar; sensitivity analyses were conducted in PQStat (version 1.8). Dichotomous outcomes were reported as odds ratios (ORs) for intention‐to‐treat and risk ratios (RRs) for adverse events, both with 95% confidence intervals (CIs). A random‐effects model was applied in the presence of significant heterogeneity, which was assessed using the chi‐square and *I* ^2^ tests. Heterogeneity was considered significant if *I* ^2^ > 50%, and the chi‐square test yielded *p* < 0.1, as per Cochrane Handbook (Chapter 9) recommendations (Higgins et al. 2019).

## 3 Results

### 3.1 Search Results and Study Selection

After the initial screening, 378 studies were assessed based on their titles and abstracts. After excluding 242 duplicates and 347 irrelevant studies, 31 articles advanced to full‐text screening. Ultimately, 14 RCTs were included in the final analysis (Figure 1).

**FIGURE 1:**
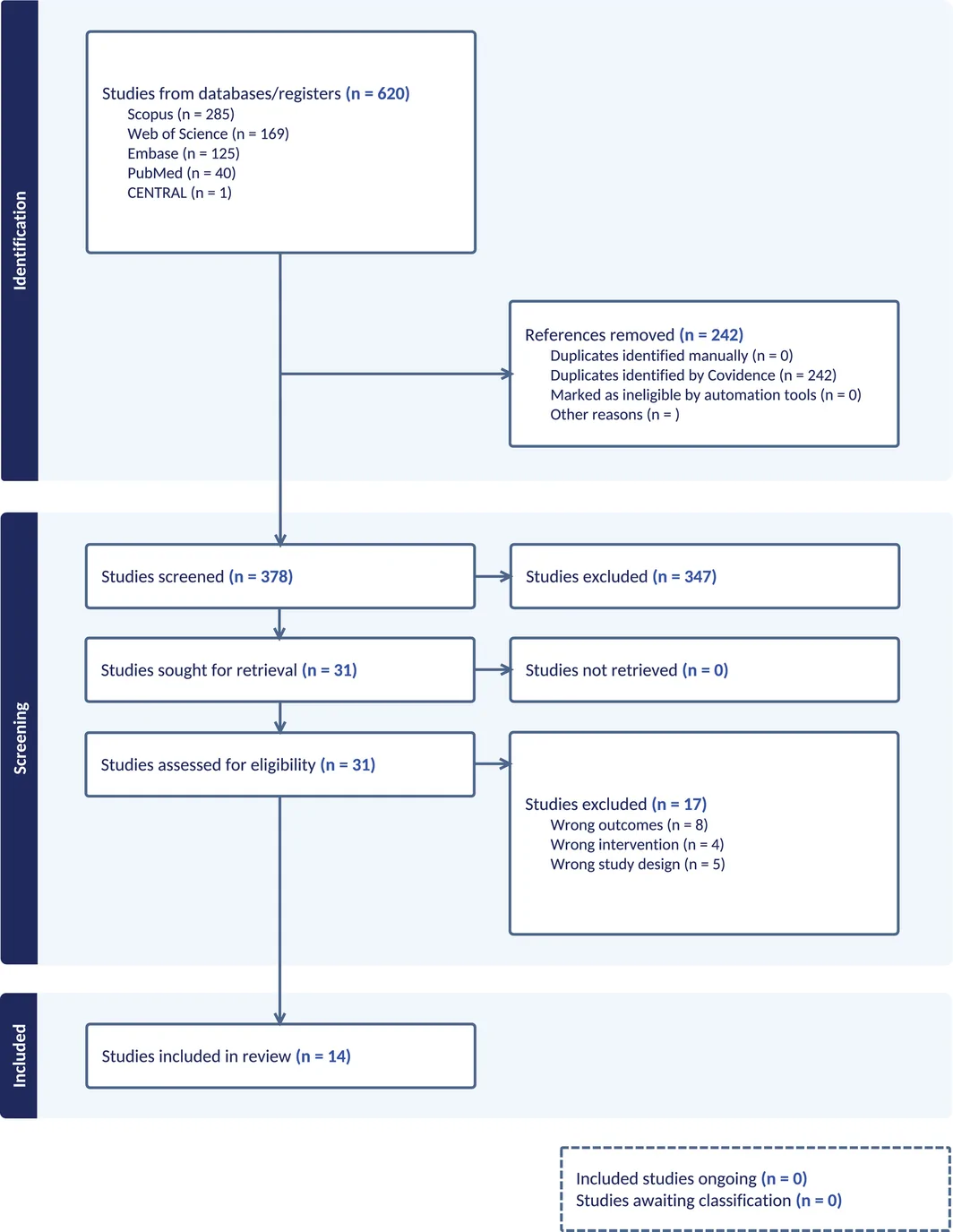
PRISMA flow chart of the screening process.

### 3.2 Characteristics of Included Studies

A total of 14 RCTs involving 1241 patients from seven countries were analysed (Dissanayake et al. 2021; Barnes et al. 2004; Dal‐Fabbro et al. 2014; De Vries et al. 2019; Glos et al. 2016; Guimarães et al. 2021; Lam et al. 2007; Ou et al. 2024; Phillips et al. 2013; Schütz et al. 2013; E. Silva et al. 2021; Uniken Venema et al. 2020, 2022; Yamamoto et al. 2019). These studies evaluated the cardiometabolic outcomes or QoL associated with MAD compared to CPAP in patients with OSA. Treatment durations varied from 4 weeks to 12 months, except for one study with a 10‐year follow‐up (Schwartz et al. 2018). Most trials enrolled adults with mild to moderate OSA. The primary outcomes varied across studies and included the ESS, 24‐h mean arterial pressure, AHI reduction, cardiovascular and metabolic parameters, quality‐adjusted life years (QALYs) and measures of autonomic function or endothelial health. Blinding was inconsistently reported, with a minority of studies using single‐blind or open‐label designs. Follow‐up durations generally matched the treatment period, with several trials extending to 12 months. Overall, the populations studied were relatively homogeneous in terms of age (range from 38 to 61 years), BMI (between 25.9 and 33.2 kg/m^2^) and disease severity; participant comorbidities, baseline characteristics and detailed RCT information are summarised in Table 2.

**TABLE 2:** Baseline characteristics of the participants.

Study ID	Characteristics								Comorbidities			
	Age (years), *m* (SD)		Male, *n* (%)		BMI, *m* (SD)		Waist circumference, *m* (SD)		HTN, *n* (%)		Diabetes, *n* (%)	
	CPAP	MAD	CPAP	MAD	CPAP	MAD	CPAP	MAD	CPAP	MAD	CPAP	MAD
Barnes et al. (2012)	47 (9.2)	47 (9.2)	83 (79.8)	83 (79.8)	31.1 (5.1)	31.1 (5.1)	NA	NA	NA	NA	NA	NA
Dal‐Fabbro et al. (2014)	47 (8.9)	47 (8.9)	NA	NA	28.4 (3.6)	28.4 (3.6)	NA	NA	9 (31)	9 (31)	2 (7)	2 (7)
De Vries et al. (2019)	NA	NA	NA	NA	30.7 (5)	29.8 (4.9)	106.8 (12.7)	106.6 (11.4)	NA	NA	NA	NA
Dissanayake et al. (2021)	49 (11)	49 (11)	73 (79)	73 (79)	30 (6)	30 (6)	NA	NA	39 (42)	39 (42)	5 (5)	5 (5)
Glos et al. (2016)	49.5 (11.8)	49.5 (11.8)	NA	NA	28.3 (4.7)	28.3 (4.7)	NA	NA	11 (69)	5 (20)	NA	NA
Guimarães et al. (2021)	49 (14)	45 (15)	17 (54.8)	15 (60.0)	28.7 (6.5)	28.2 (7.2)	100.1 (19.1)	99.4 (18.1)	NA	NA	NA	NA
Lam et al. (2007)	45 (1)	45 (2)	27 (79)	26 (76)	27.6 (3.5)	27.3 (3.5)	NA	NA	NA	NA	NA	NA
Ou et al. (2024)	60 (7.5)	61.2 (7.5)	92 (83.6)	96 (87.3)	27.7 (4)	27.8 (3.8)	97.5 (8.9)	97.8 (7.7)	110 (100)	110 (100)	65 (59.1)	65 (59.1)
Phillips et al. (2013)	49.5 (11.2)	49.5 (11.2)	102 (80.9)	102 (80.9)	29.5 (5.5)	29.5 (5.5)	101.2 (15.8)	101.2 (15.8)	NA	NA	NA	NA
Schütz et al. (2013)	38.6 (18.5)	42.3 (6.2)	9 (100)	9 (100)	25.9 (5.3)	29.3 (1.7)	NA	NA	NA	NA	NA	NA
E. Silva et al. (2021)	49 (13.6)	44.8 (15.1)	17 (55)	15 (60)	28.7 (6.5)	28.2 (7.2)	98.3 (23.7)	99.8 (19.6)	NA	NA	NA	NA
Uniken Venema et al. (2020)	59 (10)	61 (8)	17 (100)	12 (85)	33.2 (3.6)	32.4 (6.6)	NA	NA	NA	NA	NA	NA
Uniken Venema et al. (2022)	51 (9.8)	50 (9.7)	33 (79)	37 (86)	30.7 (5.3)	28.9 (4.5)	106.5 (13.1)	104.1 (7.8)	20 (48)	14 (33)	4 (10)	4 (9)
Yamamoto et al. (2019)	54.9 (12.2)	54.9 (12.2)	30 (75)	30 (75)	26.3 (3.7)	26.3 (3.7)	NA	NA	15 (37.5)	15 (37.5)	3 (8.1)	3 (8.1)

Study ID	Polysomnography							
	Apnea‐hypopnea index (AHI), *m* (SD)		Arousal index (AI), *m* (SD)		Oxygen desaturation index (both 4% and 3%), *m* (SD)		Minimum oxygen sat %, *m* (SD)	
	CPAP	MAD	CPAP	MAD	CPAP	MAD	CPAP	MAD
Barnes et al. (2012)	21.3 (13.3)	21.3 (13.3)	22 (12.2)	22 (12.2)	12.4 (15.3)	12.4 (15.3)	86.7 (6)	86.7 (6)
Dal‐Fabbro et al. (2014)	42.3 (24.3)	42.3 (24.3)	35.9 (20.5)	35.9 (20.5)	NA	NA	81.2 (5.9)	81.2 (5.9)
De Vries et al. (2019)	20.3 (6.1)	20.5 (4.6)	NA	NA	NA	NA	82.7 (4.8)	84.3 (3.9)
Dissanayake et al. (2021)	26.2 (13)	26.2 (13)	NA	NA	21.1 (12.1)	21.1 (12.1)	82.9 (7.1)	82.9 (7.1)
Glos et al. (2016)	28.5 (16.5)	28.5 (16.5)	NA	NA	21.5 (14.8)	21.5 (14.8)	NA	NA
Guimarães et al. (2021)	10.0 (4.6)	9.3 (5.2)	19.0 (12)	15.4 (13.3)	NA	NA	85.3 (6.6)	84.2 (7.3)
Lam et al. (2007)	23.8 (11)	20.9 (9.9)	21.6 (9.9)	24.5 (12.8)	NA	NA	75 (8.1)	73.8 (11)
Ou et al. (2024)	39.7 (22.6)	37.1 (19.5)	16.3 (11.9)	15.3 (13.2)	31.9 (23.4)	27 (20.6)	79.5 (7.5)	81.7 (6.8)
Phillips et al. (2013)	25.6 (12.3)	25.6 (12.3)	34.3 (15.3)	34.3 (15.3)	20.8 (12.5)	12.8 (12.5)	82.7 (7.6)	82.7 (7.6)
Schütz et al. (2013)	25.1 (10.5)	30.8 (19)	26.1 (12.6)	32.5 (12.7)	NA	NA	NA	NA
E. Silva et al. (2021)	10 (4.6)	9.3 (5.2)	19 (12)	15.4 (13.3)	NA	NA	85 (7)	84 (7)
Uniken Venema et al. (2020)	49.2 (26.1)	31.7 (20.6)	NA	NA	NA	NA	76.7 (10.1)	79.6 (6.8)
Uniken Venema et al. (2022)	20.4 (4.3)	21.1 (4.6)	NA	NA	NA	NA	82.3 (4.2)	83.6 (4.4)
Yamamoto et al. (2019)	28.6 (5.5)	28.6 (5.5)	32.9 (11.8)	32.9 (11.8)	19.3 (6.3)	19.3 (6.3)	79.6 (8)	79.6 (8)

Study ID	Blood pressure											
	24‐h mean SBP, *m* (SD)		24‐h mean DBP, *m* (SD)		Night (asleep) SBP, *m* (SD)		Night (asleep) DBP, *m* (SD)		Day (awake) SBP, *m* (SD)		Day (awake) DBP, *m* (SD)	
	CPAP	MAD	CPAP	MAD	CPAP	MAD	CPAP	MAD	CPAP	MAD	CPAP	MAD
Barnes et al. (2012)	126.5 (10.2)	126.5 (10.2)	76.3 (8.2)	76.3 (8.2)	NA	NA	69.4 (9.2)	69.4 (9.2)	NA	NA	NA	NA
Dal‐Fabbro et al. (2014)	128.6 (14)	128.6 (14)	80.6 (10.8)	80.6 (10.8)	115.9 (14)	115.9 (14)	72.7 (10.3)	72.7 (10.3)	132.5 (15.1)	132.5 (15.1)	82.8 (11.3)	82.8 (11.3)
De Vries et al. (2019)	NA	NA	NA	NA	NA	NA	NA	NA	NA	NA	NA	NA
Dissanayake et al. (2021)	125.5 (12.4)	125.5 (12.4)	79.1 (8.1)	79.1 (8.1)	113.6 (13.9)	113.6 (13.9)	69.4 (9.6)	69.4 (9.6)	129.4 (12.6)	129.4 (12.6)	82.1 (8.2)	82.1 (8.2)
Glos et al. (2016)	NA	NA	NA	NA	NA	NA	NA	NA	NA	NA	NA	NA
Guimarães et al. (2021)	118.5 (18.8)	121.4 (21)	72.1 (13.4)	74.3 (14.9)	107.1 (22.3)	110.8 (25.3)	64.9 (14.8)	66.8 (16.8)	122.2 (18.9)	125.8 (21.1)	74.5 (13.5)	77.3 (15.1)
Lam et al. (2007)	NA	NA	NA	NA	130.9 (13.9)	131.9 (16.7)	78 (11)	77.8 (12.8)	127.9 (13.3)	127.1 (15.1)	77 (10.4)	76.2 (12.2)
Ou et al. (2024)	125 (10.5)	124.8 (10.9)	79.7 (8.3)	80.5 (7.9)	121 (12)	121.8 (13.5)	77.3 (9.8)	77.7 (9)	128 (12.7)	127 (10.5)	82 (8.6)	81.3 (9.8)
Phillips et al. (2013)	NA	NA	NA	NA	NA	NA	NA	NA	NA	NA	NA	NA
Schütz et al. (2013)	NA	NA	NA	NA	NA	NA	NA	NA	NA	NA	NA	NA
E. Silva et al. (2021)	NA	NA	NA	NA	NA	NA	NA	NA	NA	NA	NA	NA
Uniken Venema et al. (2020)	NA	NA	NA	NA	NA	NA	NA	NA	NA	NA	NA	NA
Uniken Venema et al. (2022)	129.2 (10.9)	125.6 (14.2)	79.9 (7.7)	77.5 (9.8)	118.1 (10.2)	113.3 (14.7)	70.8 (7.8)	68.4 (10.3)	134.6 (11.2)	131.7 (13.8)	84.1 (8.1)	81.7 (9.9)
Yamamoto et al. (2019)	122.5 (12)	122.5 (12)	79.6 (9.1)	79.6 (9.1)	112.1 (13.1)	112.1 (13.1)	72 (10.3)	72 (10.3)	125.9 (12.1)	125.9 (12.1)	82.2 (9.3)	82.2 (9.3)

Abbreviations: BMI, body mass index; BP, blood pressure; CPAP, continuous positive airway pressure; DBP, diastolic blood pressure; HLD, hyperlipidemia; HR, heart rate; HTN, hypertension; MAD, mandibular advanced device; SBP, systolic blood pressure.

### 3.3 Risk of Bias and Certainty of Evidence

Out of the 14 RCTs included, seven demonstrated concerns for the overall risk of bias (Dissanayake et al. 2021; Barnes et al. 2004; De Vries et al. 2019; Lam et al. 2007; Phillips et al. 2013; Schütz et al. 2013; Uniken Venema et al. 2022), three exhibited a high overall risk (Dal‐Fabbro et al. 2014; Guimarães et al. 2021; E. Silva et al. 2021) and only four had a low risk of bias (Glos et al. 2016; Ou et al. 2024; Uniken Venema et al. 2020; Yamamoto et al. 2019). In Domain 1 (randomisation process), nine RCTs showed some concerns for bias (Dissanayake et al. 2021; Barnes et al. 2004; Glos et al. 2016; Guimarães et al. 2021; Lam et al. 2007; Phillips et al. 2013; Schütz et al. 2013; E. Silva et al. 2021; Uniken Venema et al. 2022), while the remaining trials were assessed as low risk (Dal‐Fabbro et al. 2014; De Vries et al. 2019; Ou et al. 2024; Uniken Venema et al. 2020; Yamamoto et al. 2019); none exhibited high risk. In Domain 2 (deviations from intended interventions), nine studies were categorised as low risk (De Vries et al. 2019; Lam et al. 2007; Ou et al. 2024; Phillips et al. 2013; Schütz et al. 2013; E. Silva et al. 2021; Uniken Venema et al. 2020, 2022; Yamamoto et al. 2019), four had some concerns (Dissanayake et al. 2021; Barnes et al. 2004; Dal‐Fabbro et al. 2014; Glos et al. 2016) and one showed a high risk of bias (Guimarães et al. 2021). Domain 3 (missing outcome data) and Domain 5 (selection of the reported result) were generally low‐risk across all studies, with two exceptions: Dal‐Fabbro et al. was assessed as high risk in Domain 3, and E. Silva et al. had some concerns. In Domain 4 (measurement of the outcome), five studies had a high risk of bias (Dal‐Fabbro et al. 2014; Guimarães et al. 2021; Phillips et al. 2013; E. Silva et al. 2021; Uniken Venema et al. 2022), five had some concerns (Barnes et al. 2004; Glos et al. 2016; Lam et al. 2007; Ou et al. 2024; Schütz et al. 2013) and four were rated as low risk (Dissanayake et al. 2021; De Vries et al. 2019; Uniken Venema et al. 2020; Yamamoto et al. 2019) (Figure 2).

**FIGURE 2:**
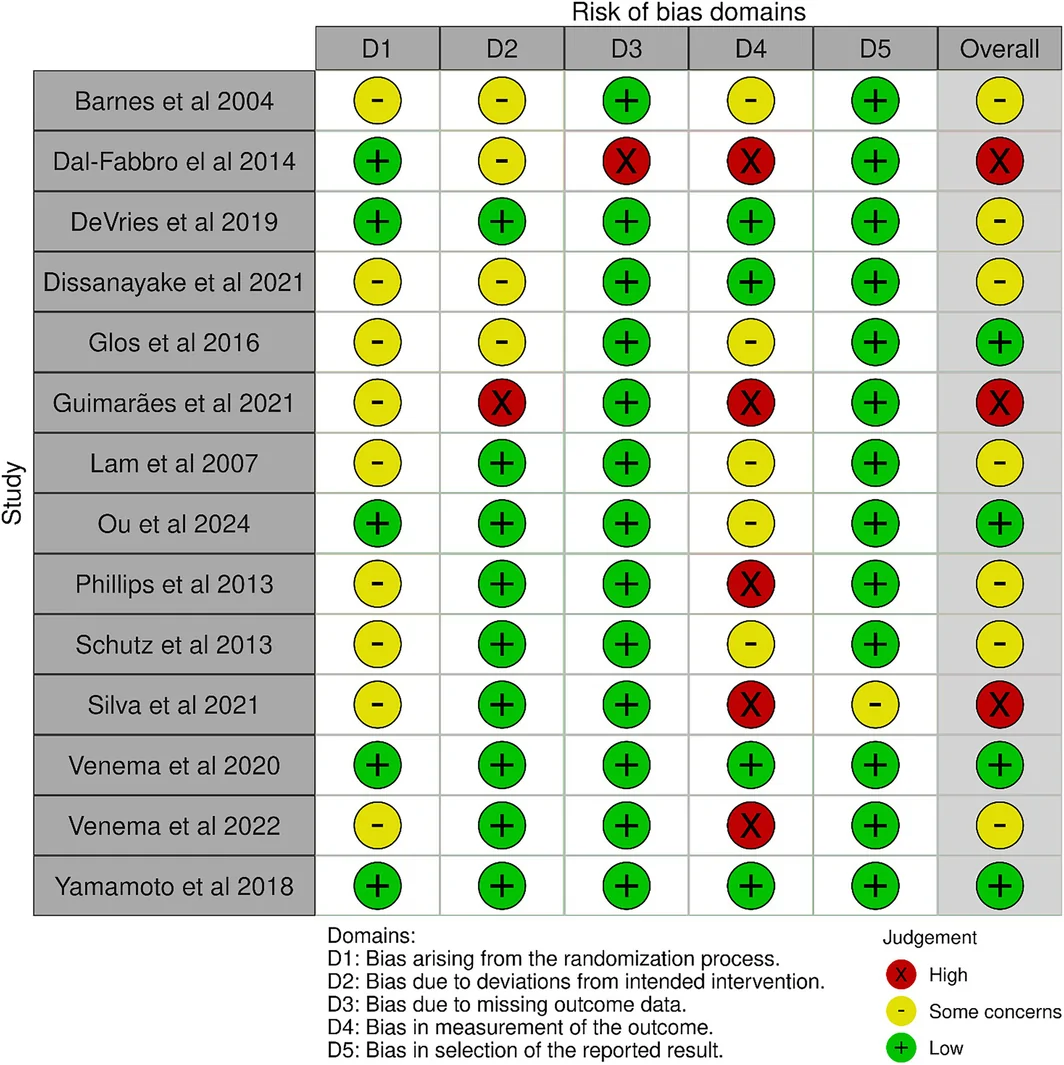
Quality assessment of risk of bias in the included trials. The upper panel presents a schematic representation of risks (low = red, unclear = yellow, and high = red) for specific types of biases of each of the studies in the review. The lower panel presents risks (low = red, unclear = yellow, and high = red) for the subtypes of biases of the combination of studies included in this review.

### 3.4 Primary Outcomes: Cardiovascular Outcomes

No significant differences were found between CPAP and MAD in 24‐h mean BP (mean difference (MD) 0.61 mmHg, 95% CI [−1.40, 2.62], *p* = 0.55), BP during wakefulness (MD 0.37 mmHg, 95% CI [−1.72, 2.47], *p* = 0.73), or asleep BP (MD 0.81 mmHg, 95% CI [−1.45, 3.07], *p* = 0.48) (Figure 3). Similarly, there were no significant differences in 24‐h mean diastolic BP (MD 0.22 mmHg, 95% CI [−1.17, 1.60], *p* = 0.76), awake diastolic BP (MD 0.01 mmHg, 95% CI [−1.39, 1.42], *p* = 0.98), or asleep diastolic BP (MD 0.14 mmHg, 95% CI [−1.30, 1.57], *p* = 0.85), except for diastolic BP dipping, which was significantly lower with CPAP (MD −3.12 mmHg, 95% CI [−5.62, −0.63], *p* = 0.01) (Figure 4).

**FIGURE 3:**
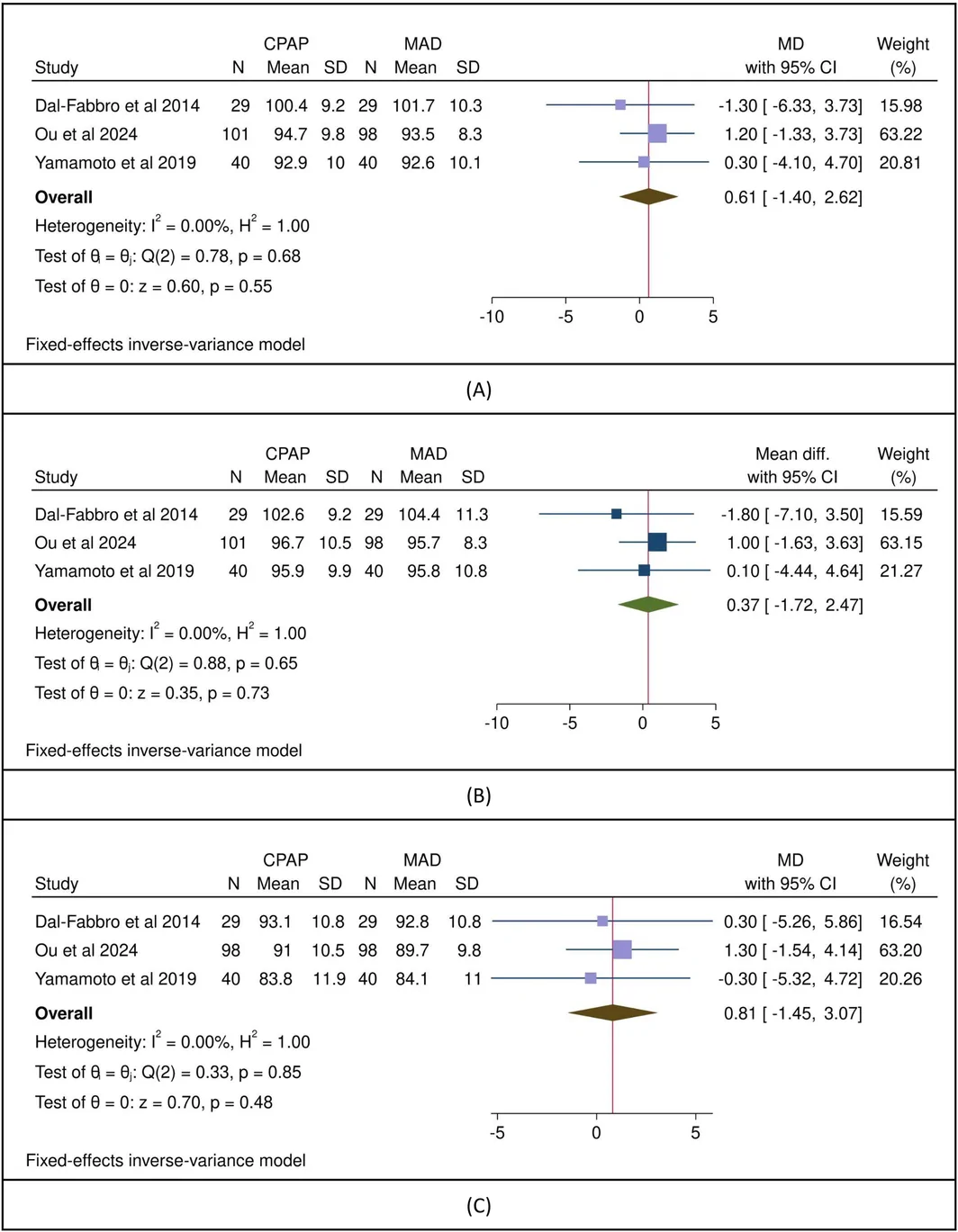
Forest plot of the efficacy outcomes. CI, confidence interval; MAP, mean arterial pressure (A: 24‐h MAP, B: awake MAP, C: sleep MAP); MD, mean difference.

**FIGURE 4:**
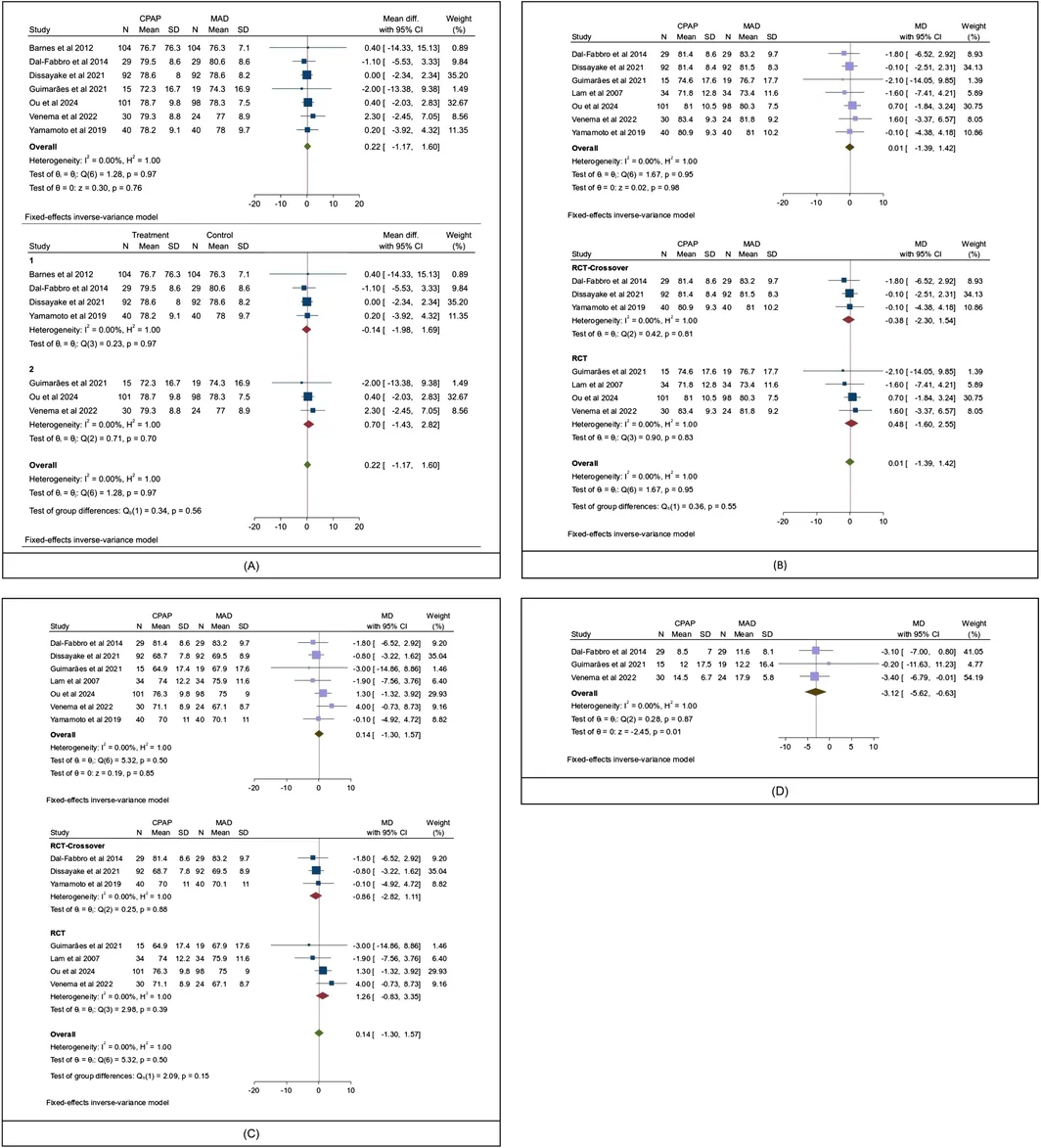
Forest plot of the efficacy outcomes. CI, confidence interval; DBP, diastolic blood pressure (A: 24‐h DBP, B: day DBP, C: night DBP, D: dipping DBP); MD, mean difference.

No significant differences were observed in 24‐h mean systolic BP (MD 0.38 mmHg, 95% CI [−1.33, 2.08], *p* = 0.67), awake systolic BP (MD 0.86 mmHg, 95% CI [−1.15, 2.87], *p* = 0.40), night systolic BP (MD 0.21 mmHg, 95% CI [−1.92, 2.34], *p* = 0.85) or systolic BP dipping (MD −0.96 mmHg, 95% CI [−3.45, 1.53], *p* = 0.45) (Figure 5). HR analysis showed no significant differences in 24‐h mean HR (MD −0.67 bpm, 95% CI [−3.28, 1.94], *p* = 0.62), HR during wakefulness (MD −1.40 bpm, 95% CI [−4.29, 1.49], *p* = 0.34) or asleep HR (MD −0.56 bpm, 95% CI [−3.05, 1.93], *p* = 0.66) (Figure S1).

**FIGURE 5:**
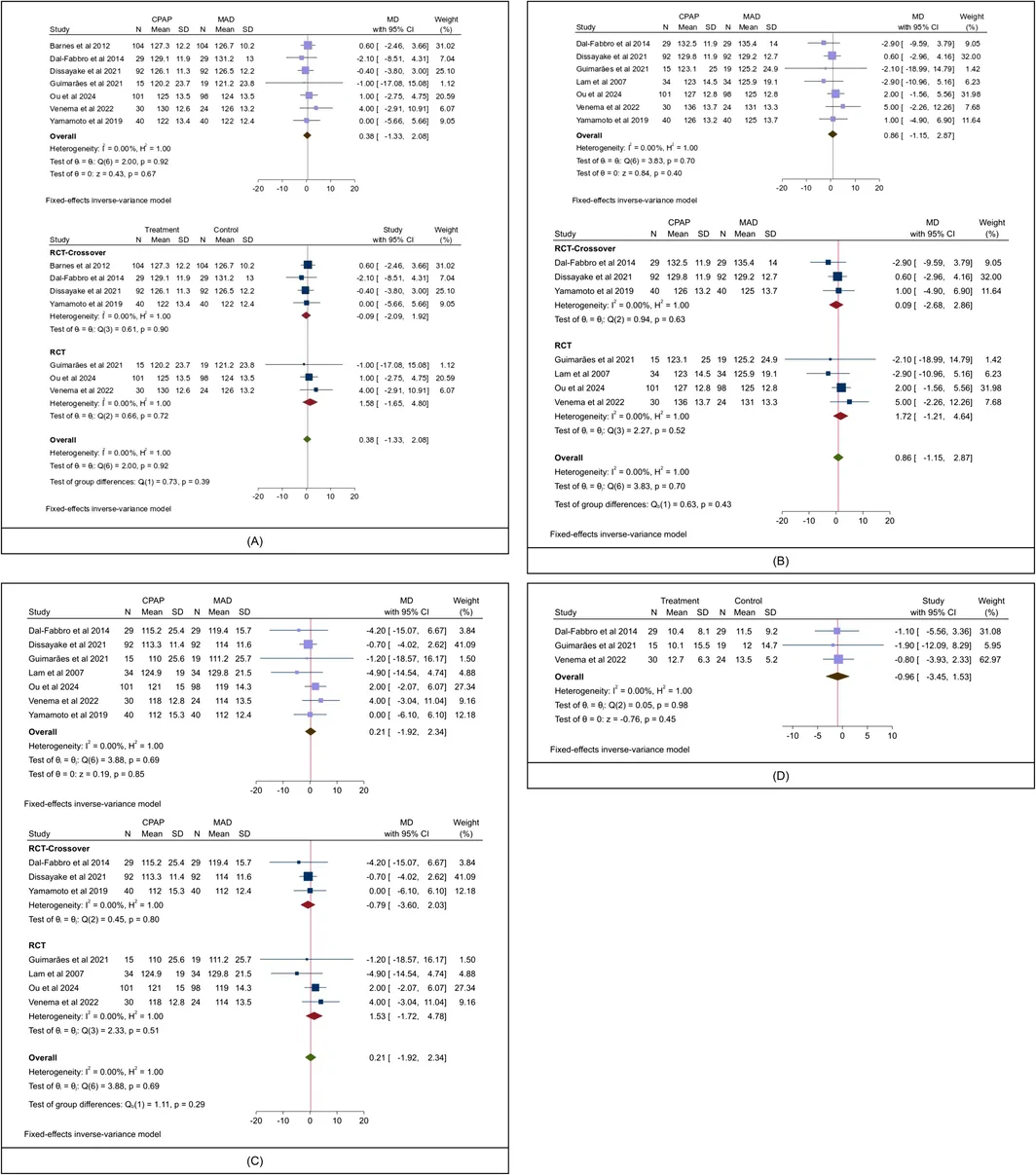
Forest plot of the efficacy outcomes. CI, confidence interval; MD, mean difference; SBP, systolic blood pressure (A: 24‐h SBP, B: day SBP, C: night SBP, D: dipping SBP).

Subgroup analysis revealed no significant differences between parallel and crossover RCTs in 24‐h mean diastolic BP (*p* = 0.56), awake diastolic BP (*p* = 0.55), night diastolic BP (*p* = 0.15), 24‐h mean systolic BP (*p* = 0.39), awake systolic BP (*p* = 0.43) or night systolic BP (*p* = 0.29). Heterogeneity across all outcomes was low (*I* ^2^ = 0%; *p* > 0.34).

### 3.5 Secondary Outcomes: QoL, Metabolic and Anthropometric Outcomes

#### 3.5.1 Anthropometric Outcomes

No significant differences were observed between CPAP and MAD regarding BMI (MD 0.56 kg/m^2^, 95% CI [−1.02, 2.14], *p* = 0.49), waist circumference (MD 2.82 cm, 95% CI [−0.81, 6.45], *p* = 0.13) and neck circumference (MD 0.42 cm, 95% CI [−0.55, 1.39], *p* = 0.40). BMI showed elevated heterogeneity (*I* ^2^ = 61.61%; *p* = 0.71), which was reduced (*I* ^2^ = 0%) after removing Schütz et al. (2013). However, this has altered the results favouring CPAP as the MAD group significantly increased BMI (MD 1.22 kg/m^2^, 95% CI [0.15, 2.28], *p* = 0.025). No heterogeneity was observed in waist circumference (*I* ^2^ = 0%; *p* = 1.00) or neck circumference (*I* ^2^ = 0%; *p* = 0.53) (Figure S2).

#### 3.5.2 QoL

ESS scores were significantly higher in the MAD group compared with CPAP (MD 0.29, 95% CI [0.19, 0.39]). The results were homogenous (*I* ^2^ = 1.24%; *p* = 0.42), and comparable results were found in subgroup analysis regardless of the study design (*p* = 0.42) (Figure S3). Concerning FOSQ, no significant differences were observed between CPAP and MAD (MD 0, 95% CI [−0.05, 0.05], *p* = 0.92), and the results were homogenous (*I* ^2^ = 39.68%; *p* = 0.17). However, there were significant differences between parallel and crossover RCTs in subgroup analysis (*p* = 0.03), as in the design of parallel RCT, FOSQ was higher in the MAD group (MD 1.22, 95% CI [0.15, 2.29]) (Figure S4).

#### 3.5.3 Metabolic Outcomes

Concerning the blood tests, the CPAP group showed significantly lower levels of LDL (MD −15.20 mg/dL, 95% CI [−28.86, −1.53], *p* = 0.03) and cholesterol (MD −17.10 mg/dL, 95% CI [−30.15, −4.05], *p* = 0.01). No significant differences were observed between CPAP and MAD regarding levels of glucose (MD 1.37 mg/dL, 95% CI [−3.17, 5.91], *p* = 0.55), HDL (MD −1.20 mg/dL, 95% CI [−8.75, 6.35], *p* = 0.76), or TG (MD −25.18 mg/dL, 95% CI [−57.11, 6.76], *p* = 0.12).

The heterogeneity was high in HDL (*I* ^2^ = 65.17%; *p* = 0.08) and was best reduced to (*I* ^2^ = 46%) after excluding Uniken Venema et al. (2022); however, the results remained consistent. No significant heterogeneity was observed in LDL (*I* ^2^ = 0%; *p* = 0.86), cholesterol (*I* ^2^ = 0%; *p* = 0.94), glucose levels (*I* ^2^ = 24.99%; *p* = 0.26) and TG (*I* ^2^ = 0%; *p* = 0.59) (Figure S5).

## 4 Discussion

CPAP and MAD have distinct mechanisms of action in the treatment of OSA. CPAP delivers a continuous stream of air through a mask, which acts as a pneumatic splint to keep the upper airway open during sleep. This positive pressure prevents airway collapse, thereby reducing apneas and hypopneas (Sériès et al. 1992). MADs, on the other hand, function by physically advancing the mandible forward. This forward positioning increases the upper airway volume, particularly in the velopharyngeal region, and reduces airway collapsibility. By displacing the tongue and soft tissues attached to the mandible, MADs help maintain airway patency during sleep. This mechanical action can effectively reduce the AHI and improve symptoms of OSA (Basyuni et al. 2018).

While CPAP is generally more effective in reducing the AHI, MAD tends to have higher compliance due to better patient comfort and preference. This leads to similar effectiveness in improving patient‐centred outcomes. Phillips et al. found that reported compliance was higher for MAD (6.5 ± 1.3 h per night) than CPAP (5.2 ± 2 h per night). This preference for MAD is also supported by Schwartz et al., who found that compliance with MAD was 1.1 h per night higher than with CPAP (Phillips et al. 2013; Schwartz et al. 2018). Long‐term adherence rates are also high for MAD. Vecchierini et al. (2021) reported that 93.3% of patients used their MAD for ≥ 4 h/night on ≥ 4 days/week at 5‐year follow‐up, with 96.5% wanting to continue MAD therapy.

This meta‐analysis compared CPAP and MAD, revealing no significant differences in BP and HR metrics, except for dipping DBP, which was lower in CPAP. CPAP also resulted in lower LDL and cholesterol levels compared to MAD. Additionally, QoL measures showed higher ESS scores for MAD, with no notable differences in FOSQ scores except in certain RCT designs. There was no significant difference between CPAP and MAD in BMI, waist and neck circumference. The American Academy of Sleep Medicine (AASM) currently recommends using MAD for adult patients with OSA who are intolerant of CPAP therapy or prefer alternate therapy (Ramar et al. 2015). However, the lack of strong evidence limits MAD utilisation in real‐world practice (Mandereau‐Bruno et al. 2021).

This study adds to previous evidence, including Bratton et al.'s network meta‐analysis, which focused on BP outcomes and found no significant difference between CPAP and MAD in reducing systolic and diastolic BP. However, both treatments were effective compared to inactive controls (Bratton et al. 2015). Schwartz et al. also compared CPAP and MAD regarding functional outcomes and QoL. They found that although CPAP was more effective in reducing AHI, there were no significant differences in quality‐of‐life measures between the two treatments. Compliance was higher with MAD, which could explain the similar effectiveness in QoL outcomes despite CPAP's higher efficacy (Schwartz et al. 2018).

Li et al. conducted a systematic review and meta‐analysis, finding that CPAP significantly reduces AHI more than MADs. However, the two groups had no significant difference in the ESS scores (Li et al. 2020). Trzepizur et al. performed an individual participant data meta‐analysis focusing on severe OSA. They concluded that while CPAP was more effective in reducing AHI and oxygen desaturation index (ODI), both treatments had similar impacts on sleep structure and patient‐centred outcomes like sleepiness and QoL. Treatment adherence was primarily in favour of MAD (Trzepizur et al. 2021). CPAP and MAD have different side effect profiles. CPAP side effects include dry mouth, nasal congestion, mask discomfort, skin irritation, pressure sores from the mask, blocked nose, nasal dryness and increased awakenings due to mask leaks or discomfort (Kalan et al. 1999; Ulander et al. 2014). MAD long‐term use can result in significant dental changes, including decreased overjet and overbite; changes in molar occlusion; TMJ pain; hypersalivation; or dry mouth can occur, affecting comfort and adherence (Uniken Venema et al. 2020; Baldini et al. 2022).

### 4.1 Limitations

This review should be interpreted considering several important limitations. Studies included patients with heterogeneous OSA severity and comorbidity profiles (including varying rates of hypertension, diabetes and metabolic syndrome), which may have influenced treatment efficacy and confounded observed treatment effects. Demographic limitations were also present, including limited ethnic diversity, predominance of male participants and a wide age range (38–61 years), restricting generalisability and potentially masking age‐specific treatment effects. Methodological constraints included variable treatment durations (4 weeks to 12 months, with only one 10‐year study), inconsistent outcome measurement methodologies and non‐standardised compliance reporting. The MAD devices used across studies varied in design, titration protocols and advancement levels, potentially affecting efficacy and patient tolerance. Our analysis lacked data on major adverse cardiovascular events (MACE) and other hard cardiovascular outcomes such as myocardial infarction, arrhythmia, or mortality, as these endpoints were not reported in the source trials; consequently, our findings are based on surrogate markers such as BP, which may not fully reflect the broader cardiovascular benefits. Several included studies demonstrated risk of bias concerns, particularly regarding blinding and randomisation processes. The inclusion of both crossover and parallel design studies introduced methodological heterogeneity, while the subjective nature of QoL measurements and variations in assessment tools limited outcome reliability. Moreover, subgroup analyses by age, sex, BMI, or sleepiness could not be performed due to insufficient data in the included trials. Given that the pooled effects were both statistically and clinically negligible, it is unlikely that meaningful subgroup trends would have emerged; nonetheless, future research should aim to explore these potential modifiers when data permit.

Despite comprehensive search strategies, publication bias cannot be ruled out. The relatively short follow‐up periods in most studies preclude definitive conclusions about long‐term comparative effectiveness of these interventions. Finally, variability in MAD titration protocols and CPAP pressure settings may have influenced treatment efficacy. Future research should address these limitations to provide more robust evidence for clinical decision‐making regarding OSA treatment options.

### 4.2 Future Implications

Future research should address the limitations identified in this meta‐analysis by examining a more diverse population, including varied ethnic backgrounds and both genders more equally, to assess the generalisability of CPAP and MAD effectiveness. Studies could also focus on long‐term outcomes beyond 12 months and evaluate hard cardiovascular outcomes. Additionally, research on optimising compliance strategies for both treatments, potentially integrating novel technologies or wearable devices, could help understand adherence behaviours better and ultimately improve treatment efficacy.

## 5 Conclusion

CPAP shows greater benefits in improving sleep metrics, some cardiovascular parameters and lipid profiles compared to MAD. MAD is associated with higher ESS but equivocal FOSQ scores. There are no significant differences between CPAP and MAD in most anthropometric metrics.

## Author Contributions

A.M. conceived the idea. A.M., M.T. and M. Abuelazm designed the research workflow. S.R. and A.K. searched the databases. S.R., A.K., A.N., M. Ayyad and Z.B. screened the retrieved records, extracted relevant data, assessed the quality of evidence and M.T. and M. Abuelazm resolved the conflicts. M. Abouzid performed the analysis. A.N., A.M., B.A. and M. Abuelazm wrote the final manuscript. M.I.A. supervised the project. All authors have read and agreed to the final version of the manuscript.

## Funding

The authors have nothing to report.

## Ethics Statement

The authors have nothing to report.

## Consent

The authors have nothing to report.

## Conflicts of Interest

The authors declare no conflicts of interest.

## Supporting information


**Table S1:** Search strategy.
**Figure S1:** Forest plots of heart rate outcomes: (A) mean heart rate, (B) mean awake heart rate, and (C) mean asleep heart rate. CI, confidence interval; MD, mean difference.
**Figure S2:** Forest plots of anthropometric outcomes: (A) body mass index (BMI), (B) BMI sensitivity analysis with omitted studies, (C) neck circumference, and (D) waist circumference. CI, confidence interval; MD, mean difference.
**Figure S3:** Forest plot of Epworth Sleepiness Scale outcomes. CI, confidence interval; MD, mean difference.
**Figure S4:** Forest plot of Functional Outcomes of Sleep Questionnaire scores. CI, confidence interval; MD, mean difference.
**Figure S5:** Forest plots of lipid and glycemic outcomes: (A) Low‐density lipoprotein cholesterol (LDL‐C), (B) total cholesterol, (C) glucose, (D) high‐density lipoprotein cholesterol (HDL‐C), and (E) triglycerides. CI, confidence interval; MD, mean difference.

## Data Availability

The data that support the findings of this study are available from the corresponding author upon reasonable request.

## References

[jsr70351-bib-0001] Baldini, N. , F. Gagnadoux , W. Trzepizur , et al. 2022. “Long‐Term Dentoskeletal Side Effects of Mandibular Advancement Therapy in Patients With Obstructive Sleep Apnea: Data From the Pays de la Loire Sleep Cohort.” Clinical Oral Investigations 26, no. 1: 863–874. 10.1007/s00784-021-04064-7 34263409

[jsr70351-bib-0002] Barnes, M. , R. D. McEvoy , S. Banks , et al. 2004. “Efficacy of Positive Airway Pressure and Oral Appliance in Mild to Moderate Obstructive Sleep Apnea.” American Journal of Respiratory and Critical Care Medicine 170, no. 6: 656–664. 10.1164/rccm.200311-1571OC 15201136

[jsr70351-bib-0003] Basyuni, S. , M. Barabas , and T. Quinnell . 2018. “An Update on Mandibular Advancement Devices for the Treatment of Obstructive Sleep Apnoea Hypopnoea Syndrome.” Journal of Thoracic Disease 10, no. S1: S48–S56. 10.21037/jtd.2017.12.18 PMC5803051 29445528

[jsr70351-bib-0004] Bratton, D. J. , T. Gaisl , A. M. Wons , and M. Kohler . 2015. “CPAP vs Mandibular Advancement Devices and Blood Pressure in Patients With Obstructive Sleep Apnea: A Systematic Review and Meta‐Analysis.” Journal of the American Medical Association 314, no. 21: 2280–2293. 10.1001/jama.2015.16303 26624827

[jsr70351-bib-0005] Dal‐Fabbro, C. , S. Garbuio , V. D'Almeida , F. D. Cintra , S. Tufik , and L. Bittencourt . 2014. “Mandibular Advancement Device and CPAP Upon Cardiovascular Parameters in OSA.” Sleep & Breathing 18, no. 4: 749–759. 10.1007/s11325-014-0937-5 24463982

[jsr70351-bib-0006] De Vries, G. E. , A. Hoekema , K. M. Vermeulen , et al. 2019. “Clinical‐ and Cost‐Effectiveness of a Mandibular Advancement Device Versus Continuous Positive Airway Pressure in Moderate Obstructive Sleep Apnea.” Journal of Clinical Sleep Medicine 15, no. 10: 1477–1485. 10.5664/jcsm.7980 PMC6778341 31596213

[jsr70351-bib-0007] Dissanayake, H. U. , J. T. Colpani , K. Sutherland , et al. 2021. “Obstructive Sleep Apnea Therapy for Cardiovascular Risk Reduction—Time for a Rethink?” Clinical Cardiology 44, no. 12: 1729–1738. 10.1002/clc.23747 PMC8715402 34791676

[jsr70351-bib-0008] E. Silva, L. O. , T. M. Guimarães , G. Pontes , et al. 2021. “The Effects of Continuous Positive Airway Pressure and Mandibular Advancement Therapy on Metabolic Outcomes of Patients With Mild Obstructive Sleep Apnea: A Randomized Controlled Study.” Sleep and Breathing 25, no. 2: 797–805. 10.1007/s11325-020-02183-1 33394328

[jsr70351-bib-0009] Francis, C. E. , and T. Quinnell . 2021. “Mandibular Advancement Devices for OSA: An Alternative to CPAP?” Pulmonary Therapy 7, no. 1: 25–36. 10.1007/s41030-020-00137-2 PMC8137783 33170490

[jsr70351-bib-0010] Glos, M. , T. Penzel , C. Schoebel , et al. 2016. “Comparison of Effects of OSA Treatment by MAD and by CPAP on Cardiac Autonomic Function During Daytime.” Sleep & Breathing 20, no. 2: 635–646. 10.1007/s11325-015-1265-0 PMC4850173 26463420

[jsr70351-bib-0011] Guimarães, T. M. , D. Poyares , L. Oliveira E Silva , et al. 2021. “The Treatment of Mild OSA With CPAP or Mandibular Advancement Device and the Effect on Blood Pressure and Endothelial Function After One Year of Treatment.” Journal of Clinical Sleep Medicine 17, no. 2: 149–158. 10.5664/jcsm.8822 PMC7853217 32964829

[jsr70351-bib-0012] Higgins, J. P. T. , J. Thomas , J. Chandler , et al. 2019. Cochrane Handbook for Systematic Reviews of Interventions. 2nd ed. John Wiley & Sons Ltd.

[jsr70351-bib-0013] Kalan, A. , G. S. Kenyon , T. A. R. Seemungal , and J. A. Wedzicha . 1999. “Adverse Effects of Nasal Continuous Positive Airway Pressure Therapy in Sleep Apnoea Syndrome.” Journal of Laryngology and Otology 113, no. 10: 888–892. 10.1017/s0022215100145517 10664702

[jsr70351-bib-0014] Lam, B. , K. Sam , W. Y. Mok , et al. 2007. “Randomised Study of Three Non‐Surgical Treatments in Mild to Moderate Obstructive Sleep Apnoea.” Thorax 62, no. 4: 354–359. 10.1136/thx.2006.063644 PMC2092459 17121868

[jsr70351-bib-0015] Li, P. , X. H. Ning , H. Lin , N. Zhang , Y. F. Gao , and F. Ping . 2020. “Continuous Positive Airway Pressure Versus Mandibular Advancement Device in the Treatment of Obstructive Sleep Apnea: A Systematic Review and Meta‐Analysis.” Sleep Medicine 72: 5–11. 10.1016/j.sleep.2020.03.015 32534403

[jsr70351-bib-0016] Mandereau‐Bruno, L. , D. Léger , and M. C. Delmas . 2021. “Obstructive Sleep Apnea: A Sharp Increase in the Prevalence of Patients Treated With Nasal CPAP Over the Last Decade in France.” PLoS One 16, no. 1: e0245392. 10.1371/journal.pone.0245392 PMC7802947 33434230

[jsr70351-bib-0017] Ou, Y. H. , J. T. Colpani , C. S. Cheong , et al. 2024. “Mandibular Advancement vs CPAP for Blood Pressure Reduction in Patients With Obstructive Sleep Apnea.” Journal of the American College of Cardiology 83, no. 18: 1760–1772. 10.1016/j.jacc.2024.03.359 38588926

[jsr70351-bib-0018] Page, M. J. , J. E. McKenzie , P. M. Bossuyt , et al. 2021. “The PRISMA 2020 Statement: An Updated Guideline for Reporting Systematic Reviews.” BMJ 372: n71. 10.1136/bmj.n71 PMC8005924 33782057

[jsr70351-bib-0019] Pataka, A. , S. C. Kotoulas , and P. R. Gavrilis . 2023. “Adherence to CPAP Treatment: Can Mindfulness Play a Role?” Life 13, no. 2: 296. 10.3390/life13020296 PMC9960813 36836653

[jsr70351-bib-0020] Patil, S. P. , I. A. Ayappa , S. M. Caples , R. J. Kimoff , S. R. Patel , and C. G. Harrod . 2019. “Treatment of Adult Obstructive Sleep Apnea With Positive Airway Pressure: An American Academy of Sleep Medicine Clinical Practice Guideline.” Journal of Clinical Sleep Medicine 15, no. 2: 335–343. 10.5664/jcsm.7640 PMC6374094 30736887

[jsr70351-bib-0021] Phillips, C. L. , R. R. Grunstein , M. A. Darendeliler , et al. 2013. “Health Outcomes of Continuous Positive Airway Pressure Versus Oral Appliance Treatment for Obstructive Sleep Apnea: A Randomized Controlled Trial.” American Journal of Respiratory and Critical Care Medicine 187, no. 8: 879–887. 10.1164/rccm.201212-2223OC 23413266

[jsr70351-bib-0022] Ramar, K. , L. C. Dort , S. G. Katz , et al. 2015. “Clinical Practice Guideline for the Treatment of Obstructive Sleep Apnea and Snoring With Oral Appliance Therapy: An Update for 2015: An American Academy of Sleep Medicine and American Academy of Dental Sleep Medicine Clinical Practice Guideline.” Journal of Clinical Sleep Medicine 11, no. 7: 773–827. 10.5664/jcsm.4858 PMC4481062 26094920

[jsr70351-bib-0023] Schütz, T. C. B. , T. C. A. Cunha , T. Moura‐Guimaraes , et al. 2013. “Comparison of the Effects of Continuous Positive Airway Pressure, Oral Appliance and Exercise Training in Obstructive Sleep Apnea Syndrome.” Clinics 68, no. 8: 1168–1174. 10.6061/clinics/2013(08)17 PMC3752627 24037015

[jsr70351-bib-0024] Schwartz, M. , L. Acosta , Y. L. Hung , M. Padilla , and R. Enciso . 2018. “Effects of CPAP and Mandibular Advancement Device Treatment in Obstructive Sleep Apnea Patients: A Systematic Review and Meta‐Analysis.” Sleep & Breathing 22, no. 3: 555–568. 10.1007/s11325-017-1590-6 29129030

[jsr70351-bib-0025] Sériès, F. , Y. Cormier , J. La Forge , and M. Desmeules . 1992. “Mechanisms of the Effectiveness of Continuous Positive Airway Pressure in Obstructive Sleep Apnea.” Sleep 15, no. 6: S47–S49. 1470809

[jsr70351-bib-0026] Trzepizur, W. , P. A. Cistulli , M. Glos , et al. 2021. “Health Outcomes of Continuous Positive Airway Pressure Versus Mandibular Advancement Device for the Treatment of Severe Obstructive Sleep Apnea: An Individual Participant Data Meta‐Analysis.” Sleep 44, no. 7: zsab015. 10.1093/sleep/zsab015 33493338

[jsr70351-bib-0027] Ulander, M. , M. S. Johansson , A. E. Ewaldh , E. Svanborg , and A. Broström . 2014. “Side Effects to Continuous Positive Airway Pressure Treatment for Obstructive Sleep Apnoea: Changes Over Time and Association to Adherence.” Sleep & Breathing 18, no. 4: 799–807. 10.1007/s11325-014-0945-5 24557772

[jsr70351-bib-0028] Uniken Venema, J. A. M. , M. H. J. Doff , D. S. Joffe‐Sokolova , et al. 2020. “Dental Side Effects of Long‐Term Obstructive Sleep Apnea Therapy: A 10‐Year Follow‐Up Study.” Clinical Oral Investigations 24, no. 9: 3069–3076. 10.1007/s00784-019-03175-6 31863188

[jsr70351-bib-0029] Uniken Venema, J. A. M. , G. E. Knol‐de Vries , H. Van Goor , J. Westra , A. Hoekema , and P. J. Wijkstra . 2022. “Cardiovascular and Metabolic Effects of a Mandibular Advancement Device and Continuous Positive Airway Pressure in Moderate Obstructive Sleep Apnea: A Randomized Controlled Trial.” Journal of Clinical Sleep Medicine 18, no. 6: 1547–1555. 10.5664/jcsm.9908 PMC9163609 35088708

[jsr70351-bib-0030] Vecchierini, M. F. , V. Attali , J. M. Collet , et al. 2021. “Mandibular Advancement Device Use in Obstructive Sleep Apnea: ORCADES Study 5‐Year Follow‐Up Data.” Journal of Clinical Sleep Medicine 17, no. 8: 1695–1705. 10.5664/jcsm.9308 PMC8656912 34165074

[jsr70351-bib-0031] Yamamoto, U. , M. Nishizaka , H. Tsuda , H. Tsutsui , and S.‐I. Ando . 2019. “Crossover Comparison Between CPAP and Mandibular Advancement Device With Adherence Monitor About the Effects on Endothelial Function, Blood Pressure and Symptoms in Patients With Obstructive Sleep Apnea.” Heart and Vessels 34, no. 10: 1692–1702. 10.1007/s00380-019-01392-3 30927057

