# Supplementary material for: Continuous Positive Airway Pressure Versus Mandibular Advancement Devices Impact on Cardio‐Metabolic Outcomes in Patients With Obstructive Sleep Apnea: A Systematic Review and Meta‐Analysis of Randomised Controlled Trials
Source: J Sleep Res. 2026 Jun 24;35(5):e70351. doi: 10.1111/jsr.70351 (PMC13619073; doi:10.1111/jsr.70351)
Supplement: Supplementary file 1 [file JSR-35-e70351-s001.docx]

**Title.**

Continuous Positive Airway Pressure Versus Mandibular Advancement Devices Impact on Cardio-metabolic Outcomes in Patients with Obstructive Sleep Apnea: A Systematic Review and Meta-analysis of Randomized Controlled Trials

**Running Title.**
CPAP vs. MAD: Cardiometabolic Outcomes in OSA.

**Authors.**

AlMothana Manasrah^1^, Mohammad Tanashat^2^, Ramy Ghaly^3^, Mohamed Abouzid^4^, Shrouq Ramadan^5^, Abdo-rhman Khassawneh^6^, Abubakar Nazir^7^, Zaid Bataineh^8^, Mohammad Ayyad^9^, Mohamed Abuelazm^10^, Basel Abdelazeem^11^, Muhammad Imran Ali^1^

**Affiliations.**

1. Department of Pulmonology and Crtical Care, United Health Services - Wilson Medical Center, Johnson City, NY, USA (AM: [Almothanamanasrah@gmail.com](mailto:Almothanamanasrah@gmail.com); MIA: [imranali175@gmail.com](mailto:imranali175@gmail.com))
2. Faculty of Medicine, Yarmouk University, Irbid, Jordan ([Mohammad_tanashat@hotmail.com](mailto:Mohammad_tanashat@hotmail.com))
3. Department of Internal Medicine, University of Missouri-Kansas City, Kansas City, MO, USA ([ghalyr@umkc.edu](mailto:ghalyr@umkc.edu))
4. Department of Physical Pharmacy and Pharmacokinetics, Poznan University of Medical Sciences, Poznan, Poland ([mmahmoud@ump.edu.pl](mailto:mmahmoud@ump.edu.pl))
5. Internal Medicine Department, AinShams University, Cairo, Egypt ([shroukm.ramadan@gmail.com](mailto:shroukm.ramadan@gmail.com))
6. Internal Medicine Department, Henry Ford Macomb Hospital, Clinton Twp, MI, USA ([Abdorahmankhassawneh@gmail.com](mailto:Abdorahmankhassawneh@gmail.com))
7. Internal Medicine Department, King Edward Medical University, Lahore, Pakistan ([abu07909@gmail.com](mailto:abu07909@gmail.com))
8. Faculty of Medicine, Jordan University of Science and Technology, Irbid, Jordan ([zaid.bataineh23@gmail.com](mailto:zaid.bataineh23@gmail.com))
9. Internal Medicine Department, Rutgers New Jersey Medical School, Newark, NJ, USA ([dr.mohammad.ayyad@gmail.com](mailto:dr.mohammad.ayyad@gmail.com))
10. Faculty of Medicine, Tanta University, Tanta, Egypt
    ([dr.mabuelazm@gmail.com](mailto:dr.mabuelazm@gmail.com))
11. Cardiology Department, West Virginia University, Morgantown, WV, USA ([baselelramly@gmail.com](mailto:baselelramly@gmail.com))

Content

**Tables**

Table S1: Search strategy.

**Supplemental Figure Legends**

Figure S1. Forest plots of heart rate outcomes: (A) Mean heart rate, (B) Mean awake heart rate, and (C) Mean asleep heart rate. MD, mean difference; CI, confidence interval.

Figure S2. Forest plots of anthropometric outcomes: (A) Body mass index (BMI), (B) BMI sensitivity analysis with omitted studies, (C) Neck circumference, and (D) Waist circumference. MD, mean difference; CI, confidence interval.

Figure S3. Forest plot of Epworth Sleepiness Scale outcomes. MD, mean difference; CI, confidence interval.

Figure S4. Forest plot of Functional Outcomes of Sleep Questionnaire scores. MD, mean difference; CI, confidence interval.

Figure S5. Forest plots of lipid and glycemic outcomes: (A) Low-density lipoprotein cholesterol (LDL-C), (B) Total cholesterol, (C) Glucose, (D) High-density lipoprotein cholesterol (HDL-C), and (E) Triglycerides. MD, mean difference; CI, confidence interval.

**Tables**

Table S1: Search strategy.

| Database | Search strategy | Results |
| --- | --- | --- |
| WOS (articles) | (("obstructive sleep apnea" OR "OSA " OR "obstructive sleep apnea syndrome" OR ”OSAHS” OR “upper airway resistance sleep apnea syndrome”)) AND (("continuous positive airway pressure" OR "positive airway pressure therapy" OR "CPAP")) AND ((“mandibular advancement devices” OR “mandibular advancement splints” OR “mandibular advancement appliances” OR “mandibular repositioning appliances” OR “dental night guards” OR “occlusal splint”)) | 169 |
| Embase (Articles) | (("obstructive sleep apnea" OR "OSA " OR "obstructive sleep apnea syndrome" OR ”OSAHS” OR “upper airway resistance sleep apnea syndrome”)) AND (("continuous positive airway pressure" OR "positive airway pressure therapy" OR "CPAP")) AND ((“mandibular advancement devices” OR “mandibular advancement splints” OR “mandibular advancement appliances” OR “mandibular repositioning appliances” OR “dental night guards” OR “occlusal splint”)) | 125 |
| Scopus (articles) | (("obstructive sleep apnea" OR "OSA " OR "obstructive sleep apnea syndrome" OR ”OSAHS” OR “upper airway resistance sleep apnea syndrome”)) AND (("continuous positive airway pressure" OR "positive airway pressure therapy" OR "CPAP")) AND ((“mandibular advancement devices” OR “mandibular advancement splints” OR “mandibular advancement appliances” OR “mandibular repositioning appliances” OR “dental night guards” OR “occlusal splint”)) | 285 |
| PubMed (RCT and clinical study) | (("obstructive sleep apnea" OR "OSA " OR "obstructive sleep apnea syndrome" OR ”OSAHS” OR “upper airway resistance sleep apnea syndrome”)) AND (("continuous positive airway pressure" OR "positive airway pressure therapy" OR "CPAP")) AND ((“mandibular advancement devices” OR “mandibular advancement splints” OR “mandibular advancement appliances” OR “mandibular repositioning appliances” OR “dental night guards” OR “occlusal splint”)) | 40 |
| CENTRAL | (("obstructive sleep apnea" OR "OSA " OR "obstructive sleep apnea syndrome" OR ”OSAHS” OR “upper airway resistance sleep apnea syndrome”)) AND (("continuous positive airway pressure" OR "positive airway pressure therapy" OR "CPAP")) AND ((“mandibular advancement devices” OR “mandibular advancement splints” OR “mandibular advancement appliances” OR “mandibular repositioning appliances” OR “dental night guards” OR “occlusal splint”)) | 1 |

Figure S1. Forest plots of heart rate outcomes: (A) Mean heart rate, (B) Mean awake heart rate, and (C) Mean asleep heart rate. MD, mean difference; CI, confidence interval.

Figure S2. Forest plots of anthropometric outcomes: (A) Body mass index (BMI), (B) BMI sensitivity analysis with omitted studies, (C) Neck circumference, and (D) Waist circumference. MD, mean difference; CI, confidence interval.

Figure S3. Forest plot of Epworth Sleepiness Scale outcomes. MD, mean difference; CI, confidence interval.

Figure S4. Forest plot of Functional Outcomes of Sleep Questionnaire scores. MD, mean difference; CI, confidence interval.

Figure S5. Forest plots of lipid and glycemic outcomes: (A) Low-density lipoprotein cholesterol (LDL-C), (B) Total cholesterol, (C) Glucose, (D) High-density lipoprotein cholesterol (HDL-C), and (E) Triglycerides. MD, mean difference; CI, confidence interval.

|  |  |
| --- | --- |
| (A) | (B) |
|  |  |
| (C) | (D) |
|  | |
| (E) | |
